# Lesion of Aggregated Monocytes and Mesothelial Cells: Mesothelial/Monocytic Incidental Cardiac Lesion

**DOI:** 10.1155/2013/836398

**Published:** 2013-03-27

**Authors:** Hilal Erinanç, Murat Günday, Tonguç Saba, Mehmet Özülkü, Atilla Sezgin

**Affiliations:** ^1^Department of Pathology, Faculty of Medicine, Başkent University, Hocacihan Mahallesi Saray Caddesi No. 1, Selcuklu, 42080 Konya, Turkey; ^2^Department of Cardiovascular Surgery, Başkent University, Ankara, Turkey

## Abstract

A 58-year-old woman with a history of childhood acute rheumatic fever and resultant mitral valve stenosis was admitted to our cardiovascular surgery clinic complaining of tachycardia, dyspnea, and chest pain. After clinical and radiological findings were evaluated, mitral valve replacement, tricuspid De Vega annuloplasty and plication, and resection of giant left atrium were performed. Atrial thrombus was removed from the top of the left atrial wall. Operation material considered as thrombus was sent to a pathology laboratory for histopathological examination. It was diagnosed with mesothelial/monocytic incidental cardiac lesion (cardiac MICE). Microscopic sections revealed that morphological features of the lesion were different from thrombus. The lesion was composed of a cluster of histiocytoid cells with abundant cytoplasm and oval shaped nuclei and epithelial-like cells resembling mesothelial cells within a fibrin network. Epithelial-like cells formed a papillary configuration in the focal areas. Mitotic figures were absent. Here we present a case which was incidentally found in a patient who underwent mitral valve replacement surgery, as a thrombotic lesion on the left atrium wall.

## 1. Introduction

Mesothelial/monocytic incidental cardiac excrescence, (cardiac MICE), is a benign lesion which was diagnosed incidentally in cardiac chambers, valves, and pericardial sac. It is a rare entity composed of mesothelial cells forming tubules, micropapillary structures and cordons, inflammatory cells, and histiocytes. Although histopathogenesis of the lesion is still unclear, some theories have been proposed to explain how the mesothelial cells exist in the cardiac chambers and valves.

Here a case called “cardiac MICE” which was incidentally found in a patient who underwent mitral valve replacement surgery, as a thrombotic lesion on the left atrium wall, is presented.

## 2. Case Report

A 58-year-old woman with a history of childhood acute rheumatic fever and resultant mitral valve stenosis was admitted to our cardiovascular surgery clinic complaining of palpitation, dyspnea, and chest pain. Hepatomegaly and 3/6 systolic murmur on mitral focus were in her physical examination. An electrocardiogram showed atrial fibrillation in all derivations. A transesophageal cardiac echocardiogram showed severe mitral valve stenosis, tricuspid insufficiency and thrombus measuring 5 cm in diameter and having calcification areas on the superior wall of left atrium. The left atrium was significantly dilated and it measured 17 × 13 cm in diameter. The laboratory findings were in normal limits. After clinical and radiological findings were evaluated, mitral valve replacement, tricuspid De Vega annuloplasty and plication, and resection of giant left atrium were performed. Atrial thrombus was removed from the top of the left atrial wall. Operation material which was considered as thrombus was sent to a pathology laboratory for histopathological examination. 

**Figure 1:**
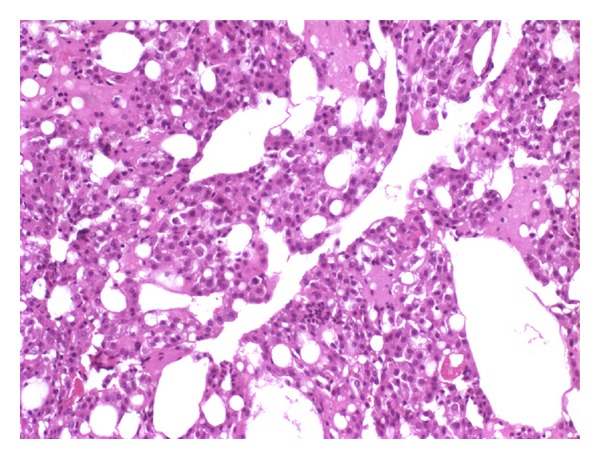
Picture shows epithelial cells arranged in strands and gland-like structures with eosinophilic cytoplasm and small dark staining nuclei.

Macroscopically, the lesion was 5 × 1, 5 × 1 cm diameter, gelatinous, soft in consistency, brown colored, and with hemorrhagic appearance. Microscopic sections revealed that morphological features of the lesion were different from thrombus. The lesion was composed of a cluster of histiocytoid cells with abundant cytoplasm and oval shaped nuclei and epithelial-like cells resembling mesothelial cells within a fibrin network. Epithelial-like cells formed papillary configuration in the focal areas (Figure 1). Mitotic figures were absent.

**Figure 2:**
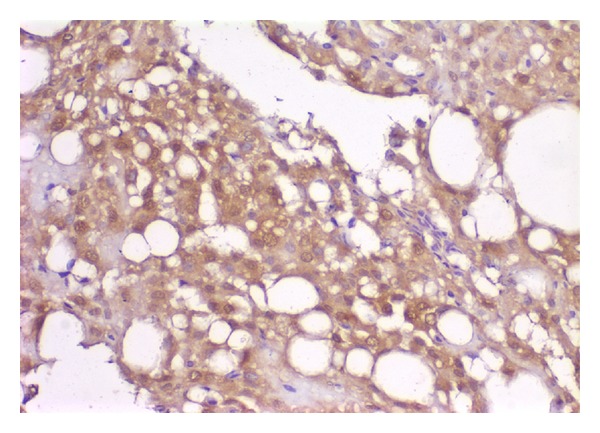
Picture shows CK5/6 positive cells in the lesion (this antibody was used to search for a mesothelial origin).

The epithelial strips were composed of cuboidal-to-low columnar cells, showing strong membranous immunostaining for cytokeratin AE1/AE3 and cytokeratin 5/6 (Figure 2). Mesothelial origin was supported by the CK5/6 expression. The histiocytic component showed intense cytoplasmic immunostaining for CD68.

## 3. Discussion

Mesothelial/monocytic incidental cardiac excrescence (MICE) is a small nonneoplastic clot-like lesion composed of mesothelial cells, inflammatory cells, adipocytes, and fibrin without a vascular network or supporting stroma [1]. 

Although thrombus and vegetation are the most common lesions that are considered in the differential diagnosis of intracardiac lesions, cardiac MICE is a very rare lesion. It has been mentioned that until recently 35 cases of cardiac MICE were reported in English medicine literature [2]. In the largest series including 14 cases of cardiac MICE, reported by Luthringer et al., it is remarked that in ten of thesecases it was seen in the endocard, in one case it was within an ascending aorta, and in three cases it was found in the pericardial sac [3]. All of these lesions were small and have been found incidentally during the surgery. In their report, Luthringer et al. have suggested that mesothelial cells migrate through the cardiac wall at the site of perforation during cardiac catheterization. This explanation has been called “reactive theory” and many other reports which emphasized the relation between cardiac catheterisation and cardiac MICE have been published.

In their report, Courtice et al. have pointed out different mechanisms which caused mesothelial proliferation. Courtice et al. demonstrated that the materials obtained from the extracorporeal bypass pump filters and mediastinal drains had similar histological features to cardiac MICE and they remarked that this material could be transferred into the cardiac chambers by the suction catheter tips during the surgery [4]. This explanation has been called “iatrogenic” or “artificial” theory. This theory is also acceptable and has been proposed by many researchers [5, 6]. 

Although these theories explain the pathogenesis of cardiac MICE, some cases without a history of prior cardiac instrumentation and prior to any surgical or invasive manipulation have also been reported [7]. With regard to etiopathogenesis, cardiac MICE formation in the patient mentioned before has not yet been elucidated. Some authors have speculated that cardiac MICE may be just a reactive lesion which results from inflammation or tumor [8]. 

 In the literature, some published cases which supported this theory were also seen. Argani et al. reported a first case of cardiac MICE in the pericardial sac associated with lung adenocarcinoma. In their case, the patient had no history of prior cardiac surgery or catheterization. The authors indicated that the possible mechanism of cardiac MICE could be prothrombogenic activity of invasive adenocarcinoma [9]. 

 Similarly, organizing pneumonia was hypothesized to have led to the formation of two pleural lesions with similar histology to cardiac MICE [10]. Morphologically, similar lesions to cardiac MICE have also been reported in different localisations such as pleural and abdominal cavities [11, 12]. In 1975, Rosai and Dehner described a first series in hernia sacs, calling the lesion nodular mesothelial hyperplasia [13]. Because of the morphological similarities to cardiac MICE, authors have considered that both lesions may have the same etiopathogenesis.

Recently, authors have pointed out that aberrant expression of cell-to-cell adhesion molecules may be related to the development of aggregates of mesothelial cells and histiocytes especially for nodular mesothelial hyperplasia. Suarez-Vilela and İzquierdo-Garcia reported that CD34 on mesothelial cells seems to be involved in adhesion cell process at cardiac MICE pathogenesis [14].

 In addition, recent studies have shown that monocytes which are the other component of the lesion can express CD 34 [15]. Monocytes-derived multipotential cells (MOMCs) derived from circulating monocytes contain progenitors capable of differentiating between different cell lineages [15, 16]. These findings may also explain how the mesothelial cells exist in the endocard. 

The importance commonly attributed to this lesion is that it may be misdiagnosed as a thrombus or neoplasia, either primary or metastatic. Through embolization, it may cause myocardial or multiorgan infarctions, in some cases it may be fatal. Although it is known that it is found incidentally, the lesion causing acute cardiopulmonary failure has also been reported [17]. 

The fact that PCNA positivity was not demonstrated in mesothelial cells supports the nonproliferative and nonneoplastic nature of the lesion [18]. Developments in cardiothoracic surgery and interventional cardiology may increase its incidence, hence the importance of recognizing this entity.

In conclusion, we report a very rare and unique case of cardiac MICE. Neither the “reactive” nor the “artifactual” theory can explain its formation. Therefore, the pathogenesis of cardiac MICE should be reevaluated and redefined in further studies. And the pathologists should be aware of this entity in examination of cardiac lesion.
